# Efficacy and safety of Xiaofeng powder (xiao feng san) in treating urticaria: A protocol for a systematic review and meta-analysis of randomized controlled trials

**DOI:** 10.1097/MD.0000000000013039

**Published:** 2018-11-09

**Authors:** Guoming Chen, Jinlong Zhao, Tengyu Chen, Zhaoping Zhang, Chuyao Huang, Zhirui Xu, Hua Xu

**Affiliations:** a Guangzhou University of Chinese Medicine; b Department of Paediatrics, First Affiliated Hospital of Guangzhou University of Chinese Medicine, Guangzhou, China.

**Keywords:** protocol, systematic review, urticaria, Xiaofeng powder

## Abstract

**Background:**

Urticaria is a common skin disease that has a high impact on a patient's daily life. Xiaofeng powder (XFP) is one of the most commonly used Chinese herbal formula in China for urticaria. However, due to the lack of systematic evaluations, its clinical efficacy remains controversial. This meta-analysis was performed to evaluate the effect and safety of XFP for urticaria.

**Methods:**

Seven databases, including Cochrane Central Register of Controlled Trials, PubMed, Embase, the Chinese National Knowledge Infrastructure (CNKI), the Chinese Scientific Journal Database (VIP), the Chinese Biomedical Literature Database (CBM), and the Wanfang Database. The period will be from their inception to September 2018. Randomized controlled trials of XFS used separately against conventional Western medicine therapy in patients with urticarial were included. After the methodologic quality was assessed and the valid data were extracted, RevMan 5.3 software was used for the final meta-analysis.

**Results:**

The results will provide evidence regarding the efficacy and safety of XFP in treating urticaria.

**Conclusion:**

The conclusion of our systematic review will provide evidence to judge whether XFP is an effective intervention for patient with urticaria. This systematic review will be disseminated in peer-reviewed publications. The results of the study will provide evidence concerning the efficacy and safety of Xiaofeng Powder (xiao feng san) in treating urticaria.

**PROSPERO registration number:**

PROSPERO CRD 42018087260.

## 1 Introduction

### 1.1 Description of the condition

Urticaria is defined as the sudden development of transient hives (wheals) and angioedema or both.^[1,2]^ Patients with urticaria may suffer itch, swellings, even the systemic anaphylaxis sometimes, which may be life-threatening. Several publications have reported that 15% to 25% among the general population may develop urticaria at some point in their lifetime.^[3–6]^ Urticaria can be further classified into specific subtypes, considering its duration, causes, and specific triggers. The acute urticaria affects 20% of the general population and the chronic form up to 5%.^[7]^ And several nationwide epidemiologic studies in German, Italy, Korea, and Taiwan also revealed that the prevalence of chronic urticaria tended to increase every year.^[8–11]^ Some surveys also showed that the quality of life of patients with urticarial is often severely affected not only because the intensity of pruritus and wheals but also impact on sleep, appetite, interpersonal relationships, and appearances.^[12–15]^

Although it is already known in ancient times, many aspects, especially with respect to the pathophysiology of various urticaria subtypes still remain unclear, in which mast cells, basophils, histamine, and other mediators may play a key role.^[16,17]^ Therefore, its optimal management remain open. Recurrently, drugs commonly used for treating urticaria include antihistamines, omalizumab, cyclosporine, and low-dose corticosteroids.^[18]^ However, up to now, the therapeutic effect on symptom control was unsatisfactory even when many kinds of these medications are used. And there has been a growing concern about side-effects and dependence on long-term medication.^[19,20]^ In consequence, it is urgent to explore alternative therapies with more satisfactory safety profiles.^[21]^ Traditional Chinese medicine (TCM) and integrated medicine are the current focus of research addressing the urticaria treatment.

### 1.2 Description of the intervention

Xiaofeng powder (XFP) is one of the widely used Chinese herbal formula in China for urticarial.^[22]^ In terms of the triggers of disease/disorders, TCM usually talks about influences that cause disharmony of human body rather than viruses or bacteria, like the Six Excesses (a generic term for the six excessive or untimely climatic influences as external pathogenic factors: wind, cold, summer-heat, dampness, dryness, and heat, which was also called “Liu Yin” in Chinese). A large amount of clinical practice experience of TCM has showed that XFP can significantly alleviate pruritic, weepy, itchy, red skin lesions over a large part of the body caused by wind-heat (a combined pathogenic factor of external wind and heat, characterized by its rapid movement, swift changes, ascending and opening actions, sensations of heat, thirst, and mental restlessness) or wind-dampness (a combined pathogenic factor of external wind and dampness, characterized by its rapid movement, swift changes, turbidity, heaviness, stickiness, and downward flowing properties) invading the body, contending with pre-existing damp-heat (a combined pathogenic factor of heat and dampness, characterized by turbid, sticky discharges, sensations of heat, thirst, and mental restlessness). The main herbs of XFP are Radix Angelicae Sinensis (dang gui), Radix Rehmanniae Glutinosae (sheng di hung), Radix Ledebouriellae Divaricatae (fang feng), Radix Anemarrhenae Asphodeloidis (zhi mu), Periostracum Cicadae (chan tui), Radix Sophorae Flavescentis (ku shen), Sesamum Indicum (hu ma), Herba Schizonepetae (jing jie), Rhizoma Atractylodis (cang zhu), Fructus Arctii (niu bang zi), Gypsum Fibrosum (shi gao), Caulis Aristolochiae Manshuriensis (mu tong), and Radix Glycyrrhizae (gan cao). Moreover, some studies have revealed that XFP has extensive influence in inhibiting inflammation, allergy, and oxidation reactions in allergic skin diseases,^[23–26]^ which may be the reasons why it is widely used and quit efficient. Therefore, our study is aimed at gathering related clinical randomized controlled trials (RCTs), to systematically evaluate its therapeutic efficacy and safety and provide the basis for clinical urticaria treatment.

## 2 Methods

This study was conducted complying with guidance from the Cochrane Handbook for Systematic Reviews of Interventions for conduct and PRISMA guidelines for reporting.^[27]^

### 2.1 Eligibility criteria

#### 2.1.1 Types of studies

All of the RCTs which reported applying Xiaofengsan to the treatment of urticaria were involved with limitations on language or publication. Trials not providing detailed information were excluded.

#### 2.1.2 Types of participants

The RCTS aimed at the patients having urticaria were involved. The participants in this study should accord with more than one of the current or past definitions of urticaria. If the trials did not expound the definitions of urticaria and TCM syndrome but just indicated that the included subjects were patients with urticaria syndrome, they were involved, too. The age, gender, or ethnic origin of the participants has no limitations.

#### 2.1.3 Types of interventions

The RCTs comparing XFS used alone vs western drugs were included. However, if trials assessed the combined effect of XFS with other interventions such as another herbal formula, qigong, TaiChi, acupuncture, moxibustion, yoga and massage, they were excluded, in consideration of that the therapeutic effect of XFS could not be distinguished. Studies that used nonconventional western medicine as control groups were also excluded. In accordance with the principle of similarity of the TCM formula,^[28]^ modified XFS should contain 4 herbs at least, and only a few herbs could be added into the XFS based on the TCM theory of syndrome differentiation. The resulting prescription should contain the following drugs: Radix Ledebouriellae Divaricatae (fang feng), Radix Angelicae Sinensis (dang gui), Radix Rehmanniae Glutinosae (sheng di huang), Periostracum Cicadae (chan tui). The number of modified herbs is limited to at most 13 (n ≤ 13.)

#### 2.1.4 Types of outcome measures

The primary outcomes were measured by total therapeutic effective rate and secondary outcomes were the proportion of patients who experienced at least one recurrence after the prophylaxis period.

### 2.2 Search strategy

The following electronic databases were systematically searched up to September 2018: Cochrane Central Register of Controlled Trials (CENTRAL), Embase, PubMed, the Chinese National Knowledge Infrastructure (CNKI), the Chinese Biomedical Literature Database (CBM), the Chinese Scientific Journal Database (VIP), and the Wanfang Database. The key search terms for literature searching wer listed as follows:): (“Urticarias” OR “Hive” OR “Urticaria”) AND (“Xiaofeng powder” OR “Eliminate Wind Powder from True Lineage” OR “xiaofeng san” OR “xiao feng san” OR “xiaofengsan”) AND (“clinical trial” OR “randomized controlled trial” OR “randomized controlled trial”). The language is limited to Chinese and English. The search strategy for PubMed is shown in Table 1.

**Table 1:**
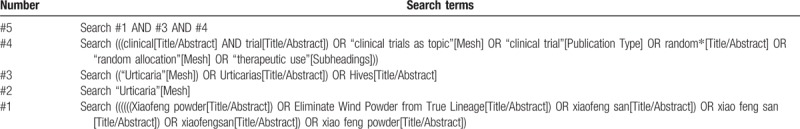
Search strategy for the PubMed database.

### 2.3 Study selection and data extraction

**Figure 1:**
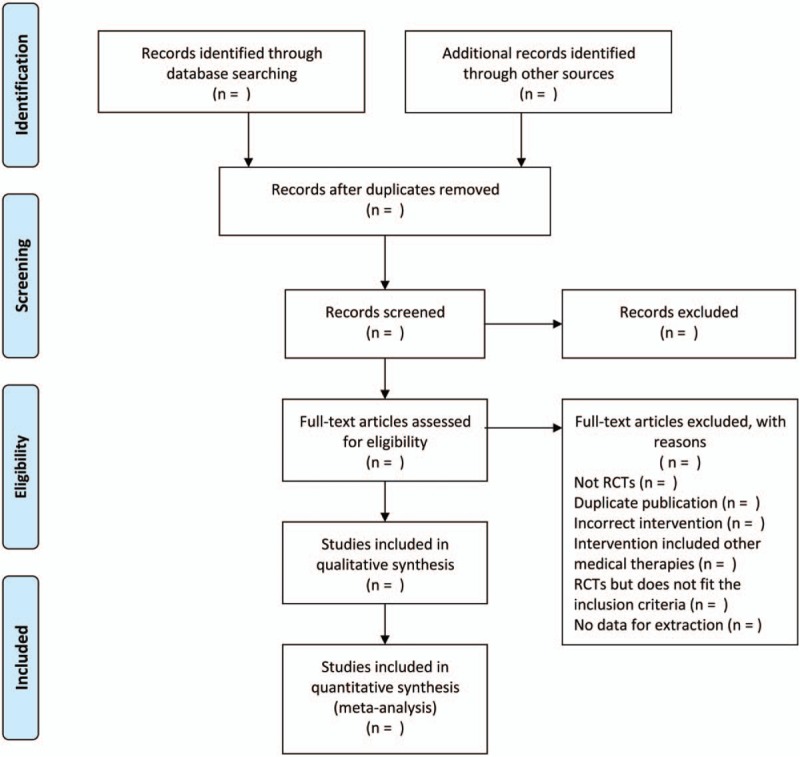
Flow diagram of study selection process.

The potentially relevant studies were first screened by 2 investigators (TC and JZ) independently according to the titles and the abstracts above. And the studies were excluded when they ran counter to the inclusion criteria or they conformed to the exclusion criteria. Duplicate papers were excluded. The discrepancies about inclusion in the meta-analysis will be talked out and resolved by the third reviewer (GC). We will use a preferred Reporting Items for Systematic Reviews and Meta-Analyses (PRISMA) flow chart to demonstrate the process of discerning, sifting out, and eliminating articles. The studies selected will be shown in a PRISMA flow chart (http://www.prisma-statement.org) (Fig. 1). Then the qualified studies were kept for further identification. The following information from primary trials were fetched in the data extraction table, mainly comprising: title; first author's name; year of publication; diagnosis standard, age, gender distribution, number of patients with urticaria, details of interventions for XFS and control groups, the composition of XFS or modified XFS, outcome measures, cointerventions, the duration of treatment, and adverse effects. Divergences were resolved by consensus between all the reviewers.

### 2.4 Addressing missing data or unclear measurement scales

If the data about the outcomes are insufficient, corresponding author for the study will be contacted by E-mail or telephone. We will only analyze the available data if enough information cannot be acquired in this way. The potential impact of missing data will be considered on the results of meta-analysis.

### 2.5 Assessment of risk of bias

The risk of bias of the involved studies was evaluated independently by 2 verifiers (TC and JZ) applying the assessment tool based on the standard in the Cochrane Handbook for Systematic Review of Interventions V.5.1.0 (updated March 2011). The constitution of the 7 items are as follow): sequence generation (selection bias); allocation concealment (selection bias); blinding of participants and personnel (performance bias); blinding of outcome assessments (detection bias); incomplete outcome data (attrition bias); selective reporting (reporting bias); and other sources of bias. Each item was assessed and classified into 3 levels: high, unclear, and low according to the above criteria. Then the methodologic quality of the trials was divided into 3 levels: low risk of bias (all items with low risk of bias), high risk of bias (at least 1 item with high risk of bias), or unclear risk of bias (at least 1 item with an unclear domain).

### 2.6 Data analysis

Studies were united based on the outcome measure, kinds of interventions, and controls. Meta-analysis was conducted using Review Manager V.5.3. According to the outcome results after treatment, the differences between the XFS and control groups could be accessed. Continuous data were analyzed using mean difference (MD), while binary data were analyzed using risk ratio (RR). Different kinds of comparisons (including XFS vs western drugs) were carried out by subgroups analysis. If trials of high quality could be discovered, there would be more comparisons between all of the studies and high-quality studies. Heterogeneity was assessed by *I*^2^ statistics. The random-effect models were used to analyze the data of Chinese medicine because it would be more exact because of its plenty of clinical heterogeneity. Funnel plots will be performed to assess reporting bias. Begg and Egger test will be used to assess the symmetry of the funnel plot and detect publication bias. The *P* values <.5 will be interpreted as statistical significance.

### 2.7 Quality of evidence

The quality of evidence of the included studies will be evaluated by the Grading of Recommendations Assessment, Development and Evaluation approach. Reviewers will take account of relevant factors including limitations of the study, inconsistencies, indirect proof, potential inaccuracies, and selective publication of positive results. Evidence quality will be divided into 4 levels: high, moderate, low, or very low.

### 2.8 Ethics and dissemination

This systematic review will be disseminated in peer-reviewed publications. The results of the study will provide evidence concerning the efficacy and safety of Xiaofeng Powder (xiao feng san) in treating urticaria. This study is a protocol for a systematic review and meta-analysis. Therefore, the approval of the Ethics Committee or Institutional Review Board was not needed. And this study does not involve patient consent.

## 3 Discussion

Xiaofeng powder is one of the Chinese herbal formulas widely applied to patients with urticaria and other dermatologic diseases. For instance, it was reported that Xiaofeng powder was the most commonly prescription used for treating urticaria in Taiwan.^[29]^ The mechanism how Xiaofeng powder reduce symptoms of urticaria as well as verifying particular observed indexes that could precisely reflect the situation of symptom relief, therefore, become the following questions. However, there is no systematic review hitherto with regard to Chinese herbal medication prescribed for urticaria. In this study, we will include relevant randomized controlled trials regarding this formula in recent years, expecting to find that Xiaofeng powder has a positive effect on treating urticaria and summarize the appropriate indicators for assessing degree of symptom relief. We aim to fill a vacancy in this area via our accomplished review.

## Author contributions

GC and HX conceived the study and designed the protocol. ZX and CH revised it. TC and JZ developed the search strategies, conducted data collection. ZZ analyzed independently. All authors have approved the final manuscript.

**Conceptualization:** Guoming Chen, Hua Xu.

**Data curation:** Jinlong Zhao, Tengyu Chen.

**Formal analysis:** Tengyu Chen, Zhaoping Zhang.

**Investigation:** Zhirui Xu.

**Methodology:** Guoming Chen, Tengyu Chen, Hua Xu.

**Project administration:** Guoming Chen, Jinlong Zhao, Hua Xu.

**Resources:** Guoming Chen, Hua Xu.

**Software:** Zhaoping Zhang.

**Supervision:** Guoming Chen, Jinlong Zhao.

**Validation:** Chuyao Huang, Zhirui Xu.

**Writing – original draft:** Guoming Chen, Jinlong Zhao, Tengyu Chen, Zhaoping Zhang, Chuyao Huang, Zhirui Xu.

**Writing – review & editing:** Hua Xu.

Hua Xu orcid: 0000-0003-2212-7347.
